# Ports in a Storm

**DOI:** 10.1289/ehp.114-a222

**Published:** 2006-04

**Authors:** Dinesh C. Sharma

The volume of global trade has been rising steadily in the past few years, fueled by free trade agreements and migration of manufacturing to destinations in Asia, particularly China. Nearly 90% of global trade is by sea, involving a fleet of 45,000 oceangoing merchant ships. U.S. ports and waterways move almost 99% of the country’s international trade by volume and 61% by value, according to *America’s Ports Today*, a February 2006 brief from the American Association of Port Authorities.

This group estimates that the volume of cargo American ports currently handle—about 2 billion tons annually—will double over the next 15 years. Chinese ports, too, are seeing a spurt in traffic. China currently has 1,430 ports and 34,000 docking berths, with a fleet of 210,000 inland and oceangoing vessels capable of handling more than 86 million tons of cargo, according to a 7 December 2005 news report from the official wire agency Xinhua. According to a 6 March 2006 report in China Daily, Shanghai is the world’s largest freight port and the third largest container port. It handled 443 million tons of cargo and more than 18 million TEUs (20-foot equivalent units) of containers in 2005.

While ports are major hubs of increasing economic activity, they are also increasingly becoming sources of local and regional pollution. And as nations around the world grapple with the health effects of shipping-related pollution, it becomes apparent that firm regulation is sorely needed.

## Sources of Pollution

For competitive economic reasons, ocean-going vessels typically use the least expensive (and often the dirtiest) fuels available. Large diesel engines propel these vessels, while auxiliary engines provide electric power for navigation, crew support, and other uses. Diesel-driven hauling equipment, trucks, and locomotives unload cargo and then ferry it to faraway inland destinations. Cruise ships idle at ports, adding to the load of diesel emissions and noise pollution.

The composition of diesel exhaust—a complex mixture of combustion products—depends on the type of engine, the speed and load at which it is run, and the composition of the fuel used. Diesel exhaust contains identified mutagens and carcinogens, and diesel exhaust particles are small enough to penetrate to the alveolar region. About 98% of the particles emitted from diesel engines are less than 10 microns in diameter (PM_10_), 94% are less than 2.5 microns in diameter (PM_2.5_),and 92% are less than 1 micron in diameter (PM_1_). As a result of incomplete combustion, the gaseous fraction also contains pollutants such as carbon monoxide, sulfur oxides (SO_x_), nitrogen oxides (NO_x_), volatile hydrocarbons, and low - molecular -weight polyaromatic hydrocarbons and their derivatives, according to the National Toxicology Program.

As one example of the potential for pollution, the Los Angeles and Long Beach ports of Southern California together are responsible for daily emissions of 128 tons of NO_x_, compared to 101 tons from all 6 million cars in the region, according to California’s South Coast Air Quality Management District. Diesel PM emissions from the combined ports were estimated at 1,760 tons per year in 2002 by the California Air Resources Board (CARB).

This is approximately 21% of the total diesel PM emissions in the South Coast Air Basin, an area that includes Orange County and portions of Los Angeles, Riverside, and San Bernardino counties. Of this, 73% was emitted by ships in coastal waters extending 14 to 100 miles offshore from California, while commercial harbor craft accounted for 14% of the total. Other sources were cargo handling equipment (10%), in-port heavy-duty trucks (2%), and in-port locomotives (1%).

A projected tripling in trade at the Los Angeles/Long Beach ports between 2005 and 2020 would result in a 50% increase in NO_x_ emissions and a 60% increase in PM from trade-related activities, if no new preventive measures are taken, according to the *Goods Movement Action Plan* released by the California Business, Transportation, and Housing Agency and the California EPA in December 2005. The plan—basically a broad statement of the problem and the state’s intention to take action to mitigate it—projects that port-related emissions are likely to account for 20% of total NO_x_ emissions in the South Coast Air Basin by 2020.

Emissions from ships engaged in international trade in the seas around Europe—the Baltic, North, Mediterranean, and Black seas, as well as the northeastern Atlantic—were estimated to be 2.6 million tons of SO_x_ and 3.6 million tons of NO_x_ a year in 2000, according to *Quantification of Emissions from Ships Associated with Ship Movements Between Ports in the European Community*, a 2002 study commissioned by the European Commission (EC). Although emissions from land-based sources are gradually coming down, those from shipping are increasing, the report said.

Dredging is a major cause of water pollution in port environments. Dredging is done routinely to create and maintain sufficient depth for safe navigation by ships. The dredged sediments are usually contaminated by industrial activities occurring in ports and through deposition of upstream sediments loaded with pollutants from other land-based sources.

Oil spills—both accidental and otherwise—also contribute greatly to water pollution. Most water pollution is a result of waste oil dumping, release of oily bilge water, washing of oil tankers (oil residue on hull walls is about 0.5% of the total load, according to the UN Environment Programme), engine operations, and the discharge of grease and oils used to maintain engines and shipboard machinery. Sometimes oily waste is illegally mixed into ship ballast water to avoid port fees. The contaminated ballast is then transferred to treatment plants that are not designed to handle the oily residue.

Ballast water itself is a cause for concern. The Global Ballast Water Management Programme of the International Maritime Organization (IMO) estimates that about 3–5 billion tons of ballast water are transferred internationally each year, often carrying exotic plant species and disease-causing organisms. A similar volume may also be transferred domestically within countries and regions each year. Invasive exotic species can alter the local ecology, affecting fisheries and threatening endangered species, besides posing a risk to human health by contaminating seafood. Fresh- and saltwater ports bear the brunt of biological invasions introduced by ballast water. Such invasions are exacerbated when exotics spread from freshwater ports into rivers and inland lakes. According to the World Wide Fund for Nature, invasive species like the North American jellyfish in the Black Sea, the mitten crab in Europe, and Asian kelp in Australia have adversely affected commercial fisheries, local species, and marine habitats. [For more on this topic, see “Exotic Invasion,” *EHP* 105:590–593 (1997)].

Still other shipping-related pollution comes from ship-breaking/salvage activities. Out-of-service ships from developed countries are sold to traders for recycling of metal scrap. “Before Bangladesh, China, India, and Pakistan became the world’s leading ship-breakers, vessels were taken apart where they were built: in industrialized countries,” points out Paul J. Bailey, a senior technical specialist with the International Labour Organization. “But high costs and environmental restrictions have driven ship owners to look elsewhere for a way of disposing of these vessels.”

The number of ships to be broken down will go up in the future, following IMO directives to phase out all single-hull oil tankers for environmental and safety purposes. In Europe alone, an estimated 2,200 ships will go out of service by the year 2010, according to Greenpeace International. About 1,800 ships from North America, Brazil, China, and other parts of Asia will go out of service in the same period.

“Ship-breakers seldom have access to basic personal protective equipment such as hard hats, gloves, and goggles for steel-cutting activities,” says Bailey. “Many are killed and thousands injured working in often torturous conditions. *Titanic*-sized vessels are floated ashore and cut up by workers who are often exposed to deadly toxicants, exploding gases, falling steel plates, and other dangers.” In the December 2005 report *End of Life: The Human Cost of Breaking Ships*, Green-peace and the International Federation of Human Rights Leagues estimated that the number of accidental deaths in ship-breaking yards of Alang in India and Chittagong in Bangladesh could exceed 100 every year. Furthermore, ships sent for breaking can contain large quantities of asbestos. Asbestos fibers were found not only at the ship-breaking yards at Alang, but in nearby living quarters, waste dumps, and places of worship. The report cites secondary data to estimate that 25% of workers in Alang will develop cancer.

Noise pollution poses further risks. Like air pollution, noise pollution can affect the cardiovascular system. Some researchers believe that air and noise pollution act synergistically. The EC has begun a project to develop a noise mapping and management system for European ports with the ultimate objective of reducing noise-related annoyance and health problems for people living around ports.

There are two aspects to the noise pollution problem. The first is the noise produced by diesel-run auxiliary engines as ships approach ports and idle at dock. In close proximity to auxiliary engines, noise levels can reach 80–120 decibels (in comparison, a chain saw averages 110 decibels). Over the past three decades, ambient noise levels in a frequency band consistent with sounds produced by large vessels have increased at a rate of about 3 decibels per decade at a single location off Southern California, according to a study published in the April 2002 issue of *Acoustics Research Letters Online*.

The second aspect is the high level of low-frequency sounds produced by vessels while cruising in the sea. These sounds can travel long distances and may change local acoustic environments, impacting marine mammals that use sound in reproductive interactions and interference with predator/prey detection. In extreme cases, noise pollution may cause habitat avoidance in these animals.

## Air Toxics: A Particular Concern

The growth in trade and resulting increase in shipping is impacting the health of workers and people living in communities near ports and major transport corridors. Air toxics, in particular, are a source of great concern.

Exposure to diesel PM_2.5_, along with secondary particles formed when sulfur dioxide (SO_2_; a form of SO_x_) and NO_x_ react with ammonia in the atmosphere, is known to cause or contribute to respiratory diseases, asthma, cardiovascular disease, lung cancer, and premature death. Emissions of NO_x_ and reactive organic gases contribute to the formation of ozone, an oxidant that can damage the respiratory tract. In 1998, the State of California listed PM emissions from diesel-fueled engines as a Toxic Air Contaminant.

Current levels of ambient air pollution in Southern California have been linked to clinically important chronic health effects, according to a May 2004 study led by John M. Peters, a professor in the Department of Preventive Medicine, University of Southern California. The report, titled *Epidemiologic Investigation to Identify Chronic Effects of Ambient Air Pollutants in Southern California*, was prepared for CARB and the California EPA. “Our findings demonstrated effects of air pollution on both new-onset asthma and asthma exacerbations. Prior to this study, the prevailing scientific view was that air pollution made existing asthma worse but that it did not cause new cases to develop,” says Peters. “We have shown that air pollution is related to bronchitic symptoms and that asthmatics are more likely to be affected than nonasthmatics.” Evaluation of the longitudinal data implicated nitrogen dioxide, PM_2.5_, and organic carbon as being responsible for the observed effects, he says.

In a study published in the 9 September 2004 issue of the *New England Journal of Medicine*, present Southern California air pollution levels were also shown to cause chronic adverse effects on lung development in children from the age of 10 to 18 years, leading to clinically significant deficits in lung function as the children reach adulthood. These deficits were associated with nitrogen dioxide, acid vapor, PM_2.5_, and elemental carbon.

Recent findings suggest that chronic health effects associated with within-city gradients in exposure to may be PM_2.5_ even larger than previously reported across metropolitan areas. In a study published in the November 2005 issue of *Epidemiology*, researchers reported observing effects nearly three times greater than those seen in earlier studies in which all the individuals within a given metropolitan area were assigned the same level of exposure based on the average ambient concentration observed at fixed points in that city. In examining specific cause of death, PM_2.5_ was associated more strongly with ischemic heart disease than with cardiopulmonary or all-cause mortality.

PM in air is also linked with post-neonatal mortality, with respiratory causes having the greatest association. An *EHP* study published online 13 January 2006 found a relationship between postneonatal mortality from respiratory causes and long-term exposure to PM_2.5_ in California (although the study did not specifically address areas affected by port-related pollution, about a third of the infants studied were born in Southern California). Among respiratory deaths, the link was stronger in low-birth-weight infants as well as those with bronchopulmonary dysplasia, as an underlying cause of death. This suggests that these infants and those infants with underlying lung conditions may be at higher risk of ill effects from air pollution.

Elsewhere, other major shipping hubs also are realizing health impacts likely due to marine emissions. In Hong Kong—home of one of the largest container ports in the world—marine emissions around Kwai Chung port are responsible for 36% of total SO_2_ pollution, compared to 6% contributed by local coal-fired power plants. This was one finding that researchers from Hong Kong University of Science and Technology and the University of California, Los Angeles, noted in a 2005 report titled *Significant Marine* Source for SO_2_ Levels in Hong Kong. “Since the health risks associated with SO_2_ and other pollutants such as PM_10_ are directly related to the concentration in which they reach sensitive receivers, the significance of the local marine sources is of considerable importance for polices to reduce the health impacts of local air pollution,” the authors wrote. “Yet, most attention has been focused on the power plant, while no controls are being imposed on the quality of fuel oceangoing cargo ships may burn while in port.”

The health risks and impacts due to shipping emissions from Shanghai’s port have yet to be assessed, though researchers have studied the impact of air quality in the city itself. According to an emission inventory prepared by the Shanghai Environmental Monitoring Center, operations at the Port of Shanghai were responsible for 44,000 tons of NO_x_, 39,000 tons of SO_x_, and 6,000 tons of PM in 2003.

“We have developed spatial distribution of emissions, covering internal creeks as well as international lines along the East China Sea [and] Yangtze River,” says Dongqing Yang, a team leader at the center. “Since the air ventilation is so good around the creeks and river, the air quality in ports is much better than the urban air quality in Shanghai. Though shipping emissions are so heavy, the air impact is not so bad around ports.”

Yang thinks the pollution effect from the Port of Shanghai is not as serious as it is in California, saying, “One major reason is large ports are located along the ocean in the estuary of the Yangtze River and East China Sea, which are far away from populated regions. People living near ports suffer more of noise rather than air pollution.”

## Understanding the Impacts

In March 2000, the South Coast Air Quality Management District published results of the second Multiple Air Toxics Exposure Study, indicating an overall average cancer risk in the South Coast Air Basin of about 1,400 per 1 million due to diesel emissions. It indicated higher risk levels in industrialized areas such as the south-central portion of Los Angeles County, freeway interchanges, and areas near air- and seaports. Now official agencies are beginning to quantify health impacts of emissions specifically related to port-related activities.

Of the 9,000 premature deaths reported annually in California from ambient levels of ozone and PM pollution, CARB attributes some 8% to emissions from ports and international goods movement, according to the board’s *Draft Emission Reduction Plan for Ports and International Goods Movement*. The draft CARB plan estimates cancer risk from diesel PM from all sources to be about 500 to 800 potential cancers per 1 million people exposed over a 70-year lifetime. A number of health effects—including heightened risk of heart disease, adverse birth outcomes, effects on the immune system, multiple respiratory effects, and neurotoxicity—were not quantified in the CARB plan due to lack of accepted burden estimates for those effects.

According to the draft plan, the largest contributors to cancer risk and other health effects are cargo-handling equipment and ships using diesel engines at dock. These emissions result in higher calculated risk due to the emissions’ proximity to residential communities. Oceangoing vessels, while under propulsion power, produce far more in the way of emissions but do not result in a comparable cancer risk since their emissions are released many miles offshore. However, these vessels’ emissions are still of considerable concern due to their potential for contributing to regional air pollution processes, says Edward Avol, a professor of clinical medicine at the University of Southern California Keck School of Medicine. These processes include photochemical formation of a number of pollutants of health concern, including ozone and PM.

CARB officials have sought peer review of their estimates of health impacts, and have identified areas in health assessment analysis that need revision. These areas include bounding estimates of health impacts of sulfates, ozone health impact assessment, and additional health end points such as chronic bronchitis.

## Not Yet There

Scientists and action groups feel that CARB’s health risk assessments are inadequate and narrow in scope. “It is difficult to measure chronic diseases epidemiologically,” says John Froines, a professor of environmental health sciences at the University of California, Los Angeles. “Given the health end points including cancer, cardiovascular disease, neurological, immunological, and developmental disorders, and allergic airway disease including asthma, it will be extremely problematic to accurately assess the true impact of expanded goods movement in coming decades on the health of exposed populations.”

The CARB assessment, he says, does not address issues such as occupational exposures, traffic accidents, psychosocial factors associated with travel, noise, and light with their implications for cardiovascular disease. Within air pollution too, the plan has not looked adequately at a range of end points that are now known to be important, nor has it attempted to quantify risks where the end points are indirect.

Official projections significantly underestimate health impacts, says Diane Bailey, an engineer with the Natural Resources Defense Council. For example, the CARB plan quantifies health impacts of shipping containers as they enter or leave an international facility, while neglecting to assess pollution impacts of containers traveling inland to distribution centers. “Future health assessments should cover all adverse public health outcomes, a wider array of pollutants known to cause adverse health impacts, and all significant sources known to emit these pollutants,” she says. “Other issues that must be discussed and fully incorporated into future analyses are cumulative risk, increased vulnerability of sensitive populations, and risks to exposed workers, besides residential populations.”

Meanwhile, the shipping industry has questioned the modeling techniques used to calculate health risks and maintains that CARB’s risk estimates are flawed. “There are flaws in the methods used by CARB and in their application. But we want to make it clear that the discussion is over the magnitude of the impacts and not over the fact that there are impacts that need to be reduced,” says T.L. Garrett, vice president of the Pacific Merchant Shipping Association. “We have some concerns on the use of modeling methods to diagnose health impacts in a population rather than use of the models to predict relative health benefits resulting from the implementation of control strategies.”

## Regulatory and Technology Issues

Health impacts, including cancer risk, have provided evidence for stronger regulations aimed at cutting shipping-related emissions. The CARB plan targets a 20% reduction in diesel PM by 2010 from 2001 levels, which it claims will reduce health risks by 60% or more by 2020. “We estimate that one dollar spent on controls saves four to eight dollars in health costs,” says Mike Scheible, deputy executive officer at CARB.

“Over half of PM_10_ and PM_2.5_, almost ninety percent of the SO_x_, and over a third of the NO_x_ emissions from port operations are traceable to oceangoing vessels,” says Avol. “Clearly, substantive reductions in this source category would have a dramatic effect on regional air quality and health effects associated with ambient levels of these pollutants.” He adds, “From the perspective of proximity to exposure and potential for noticeable improvements in local community pollution, trucks, rail, and cruise ships are significant port sources of pollution. This is because they are emitting directly in, near, or throughout the immediate community.”

In its first major regulatory step to cut shipping emissions, CARB has targeted auxiliary engines on ships. The new rule requires ships to switch over to cleaner-burning fuels in their auxiliary diesel engines and diesel–electric engines once they are within 24 nautical miles of the California coastline. Another new rule targets cargo-handling equipment such as forklifts and cranes, calling for replacing or retrofitting their engines with those using “best available control technology.” The new regulations, which come into effect 1 January 2007, are expected to cut diesel PM emissions by a total of 23,000 tons, NO_x_ emissions by 15,000 tons, and SO_x_ emissions by 200,000 tons by 2020.

“It is a good start to set goals and list possible mitigation measures, but as of yet there is really no plan or strategy,” says Bailey. “For example, we need to see commitments to specific details such as mandatory emission reduction measures rather than voluntary incentives for industry. [CARB] needs to be an active regulator of pollution in our ports and with regard to goods movement throughout California.”

The shipping industry has its own doubts about the CARB rules. Low-sulfur fuels are technically feasible and are being used by some vessels calling at West Coast ports, but switching of fuels while under way (as required under the new rule for auxiliary engines) raises operational and safety concerns, says Garrett. “The larger issue is, does the state of California have the authority to regulate international shipping beyond its traditional three-mile boundary?” he asks.

The U.S. EPA is working on reducing emissions from propulsion engines on oceangoing vessels. In 2003, the agency adopted emission standards for new Category 3 marine diesel engines installed on vessels flagged or registered in the United States from 1 January 2004 onward. Marine diesel engines differ from other diesel engines in terms of their exhaust, cooling, electrical, and fuel systems. Category 3 engines are large diesel engines used for propulsion power on container ships, tankers, bulk carriers, and cruise ships. These standards will apply until a second tier of standards for deeper emission reductions is developed; these standards should be finalized by April 2007. In the future, these standards may be made applicable to engines on foreign vessels entering U.S. ports. The EPA also intends to eventually set standards for fuels used by marine engines.

The federal agency estimates that these regulations—when fully implemented in 2030—will annually prevent up to 12,000 premature deaths, 15,000 heart attacks, and 6,000 child asthma-related emergency room visits throughout the United States.

Because issues such as engine emissions and fuel standards are international in scope, the IMO is also framing rules for cutting down shipping emissions. In May 2005, an IMO regulation on engine emission standards for NO_x_ came into force for engines above 130 kilowatts, in the form of Annex VI of the International Convention for the Prevention of Pollution from Ships. The rule includes a global cap of 4.5% by mass on sulfur content of fuel oil and recommends monitoring of sulfur content globally. (However, considering that the rolling average of sulfur content globally from 2002 through 2004 was 2.67%, the new cap may be too liberal.) The IMO is also encouraging countries to declare their coastlines as “SO_x_ Emission Control Areas” (SECAs), where sulfur content in fuel must not exceed 1.5%. The U.S. EPA is exploring a potential North American SECA.

Under the marine fuel directive adopted by the European parliament in April 2005, all ships in the Baltic SECA and passenger vessels in European Union (EU) territorial waters will have to use fuel with a 1.5% sulfur limit after 11 August 2006. The 1.5% sulfur limit will apply to the North SECA (which includes the English Channel) after 11 August 2007. The sulfur limit will be 0.1% in fuel used by passenger vessels and seagoing ships at berth in EU ports after 1 January 2010. These measures are expected to reduce shipping-related SO_2_ in the EU by over 500,000 metric tons a year from 2006.

Besides marine fuel regulation, the EC is encouraging research to assess the economic and technical feasibility of SO_x_ and NO_x_ abatement technologies such as shoreside electricity, seawater scrubbing, selective catalytic reduction, and the use of humid air motors. The EC also favors fiscal incentives and voluntary measures to encourage the use of low-sulfur fuels and green technologies by ship owners. But even after the implementation of SECAs in Europe, SO_x_ emissions from international shipping are projected to grow by 42% and NO_x_ emissions to grow by 60% by 2020. By then, emissions from international shipping around Europe will have surpassed the total from all land-based sources in the 25 member states combined, according to the 2005 report *Baseline Scenarios for the Clean Air for Europe (CAFE) Programme*.

Another emission reduction strategy is to cut idling time of vessels and tugboats by providing electric power on shore. The Port of Los Angeles has signed a lease with container terminal operator P&O Nedlloyd that would require the company to use shore power for ships at berth and alternative fuel yard tractors, and possibly employ low-sulfur fuel in vessel main engines. An additional benefit of using shoreside electricity is the elimination of noise and vibration from the auxiliary engines while they are at berth. At Sweden’s Port of Göteborg, shoreside power is sourced from wind turbines, thus foiling criticism that the use of land-sourced power is merely switching from one dirty fuel to another.

Plugging in to onshore power requires retrofitting power systems or ships, and that involves new investments; it may not be economically viable for infrequent visitors. Nearly 20% of the ships visiting California ports will use shore-based power by 2010. This number would gradually go up to 80% by 2020, according to CARB. Avol points out this is only a proposed strategy at the present time, however, and it remains to be seen how realistic it would be in practice.

Reducing the speed of vessels as they approach a port can also help cut emissions. About 70% of ships calling at the ports of Los Angeles and Long Beach participate in a voluntary speed reduction program implemented since 2002. The plan requires ships to reduce their speed from 22 knots to 12 knots or less within a 20-mile radius of the two ports. The strategy is sweetened by a financial incentive; operators qualify for a 15% discounted dockage rate during the following 12 months if 90% of their vessels comply with the 12-knot speed limit for a year.

In the first six months of 2005, speed reduction at the Port of Los Angeles saved 266 tons of NO_x_ emissions. The port now plans to extend the limit to 40 nautical miles. Authorities at the Port of Long Beach estimate that if all vessels comply with the program, the amount of NO_x_ produced by container ships would be cut by about 550 tons a year. One potential drawback to this scheme is that if ships are going to take longer to reach their destination ports, it can impact ship schedules.

Recycling of waste oil, oily bilge, and oil-contaminated waste can go a long way toward minimizing oil pollution at ports. In the 2000 report *Green Ports: Environmental Management and Technology at U.S. Ports*, researchers at the University of Massachusetts Boston Urban Harbors Institute recommended that ports provide facilities for oil collection and recycling that are easily accessible and inexpensive. Oil-dispensing facilities at ports can be encouraged to buy back used oil for recycling. Runoff from parking areas and roads that pick up oil and other wastes from land can be controlled quite effectively through filtration devices such as porous pavements, soak-away pits, and dry wells.

Main issues that need to be resolved to check shipping pollution are international and national consensus on fuel quality, emission standards, and a time frame for adoption as well as for onshore power systems. There is a need to enforce the same for other contributors to diesel emissions—cargo-handling equipment, trucks, and locomotives. For this exercise to succeed, engine makers and oil companies also need to be involved.

In many parts of the world, shipping-related emissions have already exceeded or are projected to exceed those from land-based sources in the next few years, if no reduction measures are taken. Shipping emissions can be cut substantially by deploying some of the same technologies and fuels used for cutting emissions from land-based mobile and stationary sources. But doing so poses major economic, legal, and infrastructural challenges.

The emission reduction strategies currently on the table revolve around cleaner engines, cleaner fuels, exhaust control methods, and operational programs. And a variety of mechanisms are being explored or have been proposed to implement these strategies. The feasibility of these mechanisms is being tested at various ports with varying degree of success. What is needed is expedited decision making at all levels, from IMO to port city authorities, to ensure that our ever-increasing need to trade, transfer, and transport various things around the globe doesn’t leave all of us stranded on an environmental ship of fools.

## Figures and Tables

**Figure:**
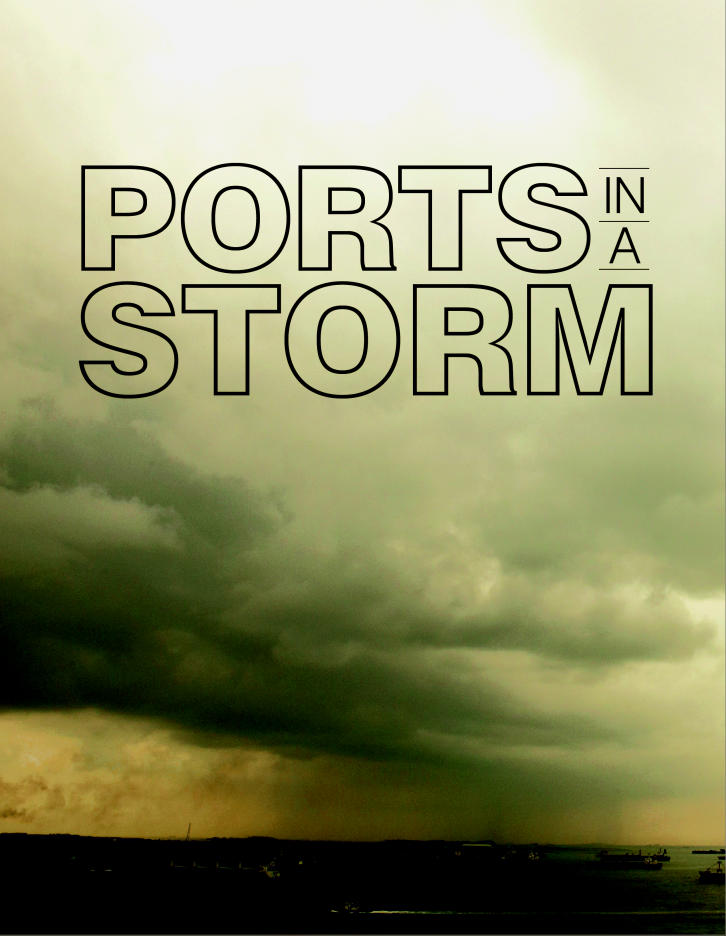


**Figure:**
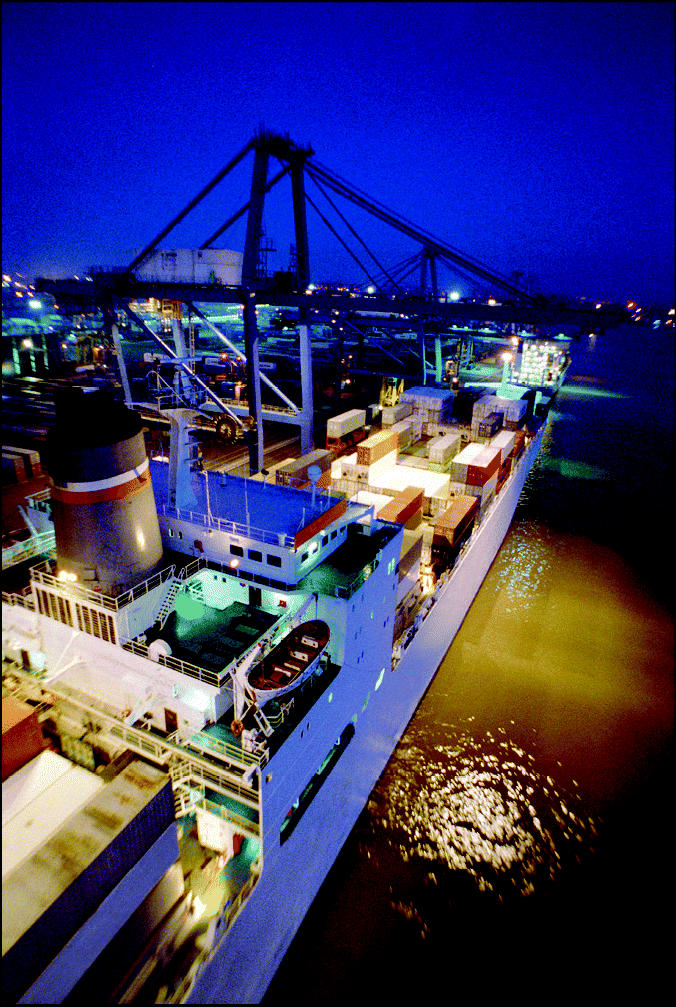
Wakeup call. Ports, long bustling centers of industry, have become centers of pollution as well.

**Figure:**
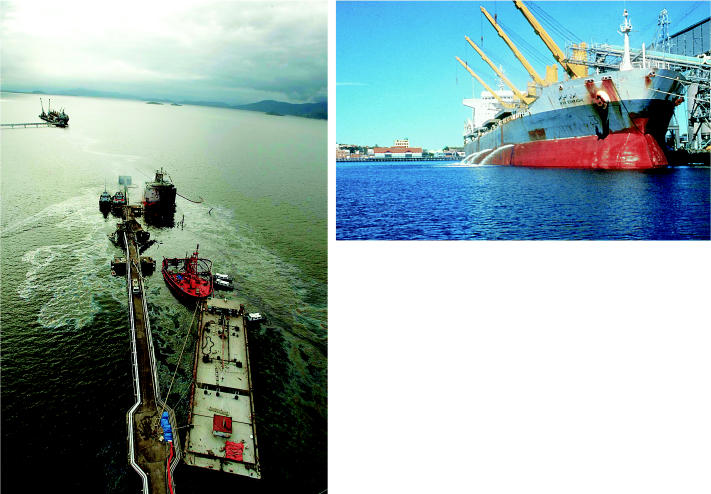
The great catch-all. (left) The Chilean cargo ship *Vicuña* exploded and broke in half while unloading ethanol at a port in Paranaguá, Brazil, in November 2004. Cleanup workers found dead fish and dolphins in the toxic slick of fuel oil, diesel fuel, and methanol that leaked from the ship. (above) Ballast water pours into harbor waters, potentially carrying pathogens, fuel contaminants, and exotic species.

**Figure:**
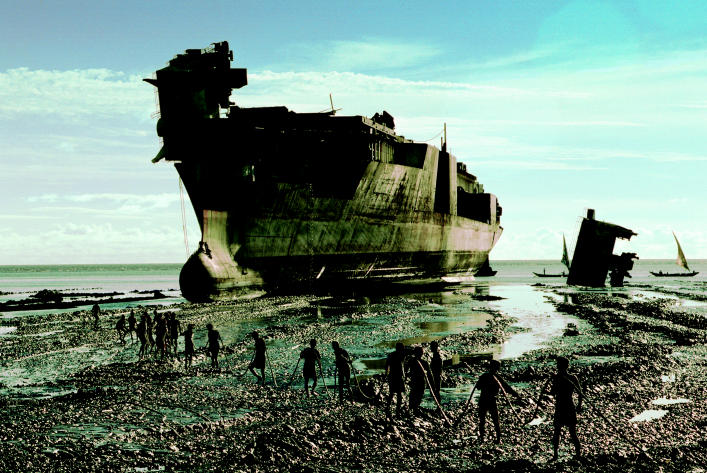
Herculean task. Ship-breakers, like these workers near Chittagong, often lack even basic personal protective equipment, and may encounter hazards such as asbestos, toxic gases, and explosions.

**Figure:**
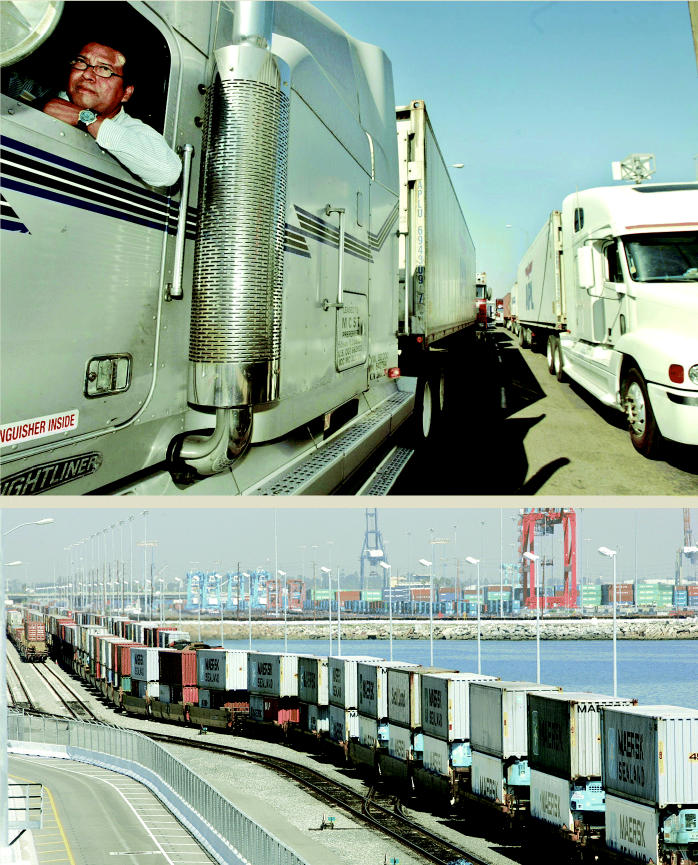
Cargo in, cargo out. (top) Truckers wait to unload their cargo at the Port of Los Angeles, adding to the burden of diesel exhaust borne by port cities. (above) Hundreds of shipping containers move in a train at the Port of Los Angeles, just one way the cargo is moved inland.

**Figure:**
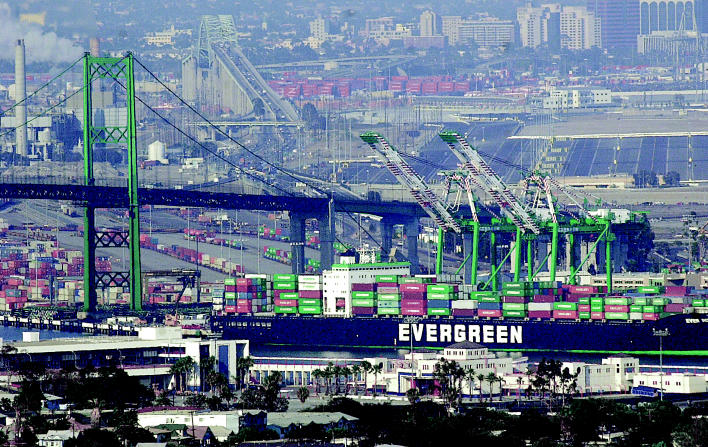
Cacophony of cargo. Noise from large vessels approaching harbor and idling at the dock adds to the din of trucks and trains arriving to transport goods. Added to the ambient noise of an urban setting, the cumulative noise can cause health effects among the population living nearby.

**Figure:**
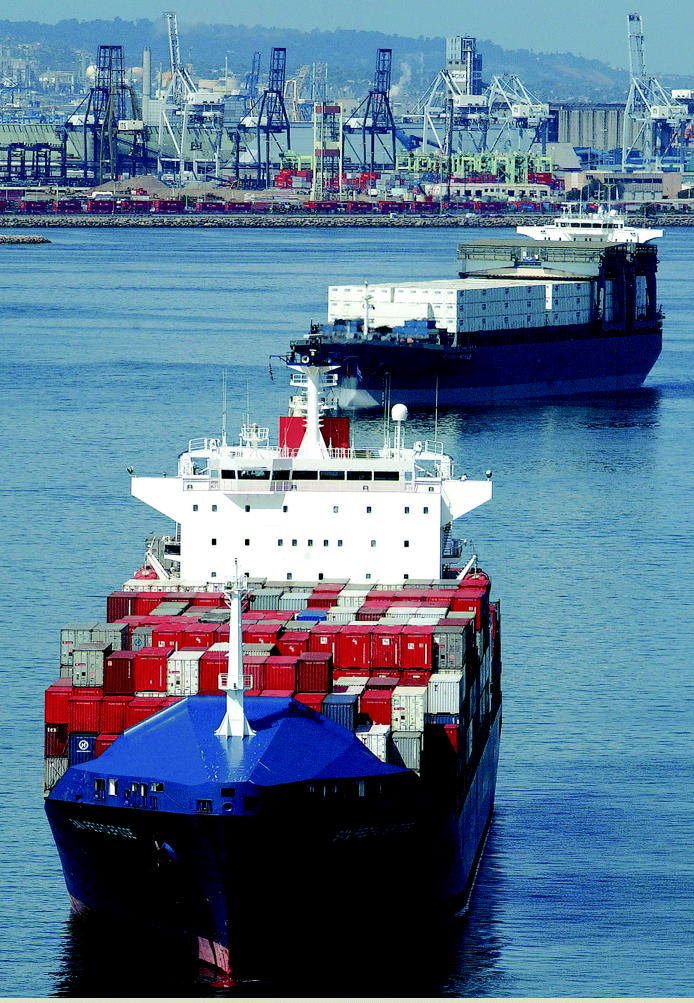
Working to contain the ill effects. A draft plan by CARB aims to lessen ships’ impact on premature deaths in California.

**Figure:**
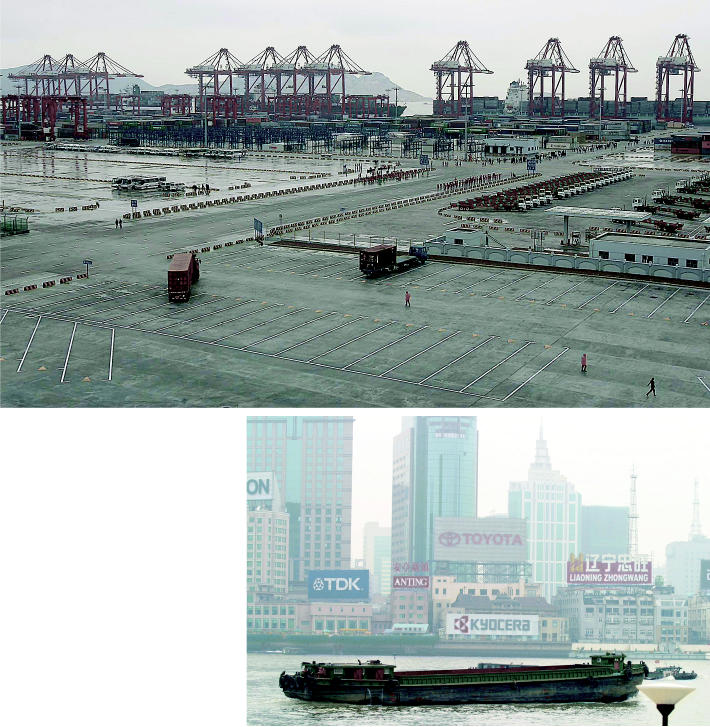
Seeking solutions in Shanghai. In December 2005, Shanghai launched operations at the Yangshan deepwater port, a mammoth facility more than 20 miles offshore in the East China Sea. A study of the spatial distribution of emissions in Shanghai’s air suggests that placement of ports farther from populated areas decreases the health effects of air pollution.

**Figure:**
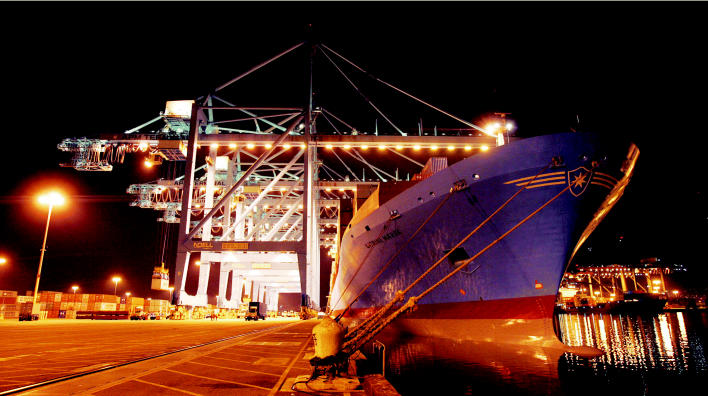
Nightowls reduce NO_x_. The twin ports of Los Angeles and Long Beach have begun operating on weekends and evenings in a new initiative designed to cut freeway congestion and emissions.

